# The Determinants of Coexisting Anemia and Undernutrition Among Pregnant Women in Southern Ethiopia: A Multi-Level Analysis

**DOI:** 10.3390/healthcare13131495

**Published:** 2025-06-23

**Authors:** Amanuel Yoseph, Lakew Mussie, Mehretu Belayineh, Ines Aguinaga-Ontoso, Francisco Guillen-Grima, G. Mutwiri

**Affiliations:** 1 School of Public Health, College of Medicine and Health Sciences, Hawassa University, Hawassa P.O. Box 5, Ethiopia; mehretu@hu.edu.et; 2 Adare General Hospital, Hawassa City Administration, Hawassa P.O. Box 5, Ethiopia; lakew365@gmail.com; 3 Department of Health Sciences, Public University of Navarra, 31008 Pamplona, Spain; f.guillen.grima@unavarra.es; 4 Group of Clinical Epidemiology, Area of Epidemiology and Public Health, Health Care Research Institute of Navarre (IdiSNA), 31008 Pamplona, Spain; 5 CIBER in Epidemiology and Public Health (CIBERESP), Institute of Health Carlos III, 46980 Madrid, Spain; 6 Department of Preventive Medicine, Clinica Universidad de Navarra, 31008 Pamplona, Spain; 7 School of Public Health, University of Saskatchewan, Saskatoon, SK S7N 5A2, Canada; george.mutwiri@usask.ca

**Keywords:** coexisting anemia and undernutrition, pregnant women, prevalence, determinants, modified Poisson regression, multi-level analysis, dietary diversity, food insecurity, Sidama region, Ethiopia

## Abstract

**Background/Objectives**: Anemia and undernutrition are severe public health concerns in Ethiopia. These are the two most common nutritional disorders in pregnant women and frequently coexist. However, to our knowledge, there is little evidence of the coexistence of anemia and undernutrition among pregnant women. Therefore, this study aimed to examine the prevalence of coexisting anemia and undernutrition (CAU) and associated factors among pregnant women. **Methods**: A community-based cross-sectional study was conducted from 1 to 25 June 2024, on 515 pregnant women in the Hawela Lida district of Sidama, Ethiopia. We utilized a multi-stage sampling method to choose eligible study participants. A pre-tested and structured questionnaire was used to collect data via the online Open Data Kit mobile tool. We controlled the effect of confounders and clustering by using a multi-level mixed-effect modified Poisson regression analysis model. **Results**: The prevalence of CAU among pregnant women was 25.4% (95% CI: 21.9–28.9). The prevalence of CAU was associated with household food insecurity (adjusted prevalence ratio [APR]: 2.17; 95% CI: 1.43–3.28), training on model family (APR: 0.66; 95% CI: 0.45–0.96), inadequate dietary diversity (APR: 1.51; 95% CI: 1.18–1.95), and having poor knowledge of nutrition (APR: 1.55; 95% CI: 1.06–2.26) at individual levels. Low community-level women’s autonomy (APR: 6.19; 95% CI: 3.42–11.22) and community-level road accessibility (APR: 0.65; 95% CI: 0.43–0.98) were the identified determinants of CAU at the community level. **Conclusions**: One in four pregnant women had CAU in the study area. Household food insecurity, inadequate dietary diversity, and poor nutrition knowledge were associated with an increased likelihood of CAU, while participation in model family training and improved road accessibility were associated with reduced CAU. We have also indicated that low community-level women’s autonomy significantly increased the risk of CAU. Therefore, inter-sectorial collaboration should be required to comprehensively address CAU’s determinants at different levels. Additionally, any CAU prevention and intervention programs should provide model family training explicitly targeting women with poor nutritional knowledge and low autonomy in healthcare decision-making.

## 1. Introduction

Undernutrition is defined as a mid-upper arm circumference (MUAC) of less than 23 cm, which is a widely accepted and validated anthropometric indicator of maternal undernutrition, particularly in low-resource settings [1,2]. Anemia during pregnancy is defined as a hemoglobin concentration below 11.0 g/dL, which is consistent with the World Health Organization classification for anemia in pregnant women [3]. Anemia is a global public health concern, affecting 36.5% of pregnant women worldwide, with a particularly high prevalence in sub-Saharan Africa and Southeast Asia [4]. Similarly, 41% of pregnant women are suffering from anemia in Ethiopia, with ranges from 9% in Addis Ababa to 65.9% in the Somali region [5]. Anemia during pregnancy is linked to negative health and socioeconomic outcomes. Pregnant women who are anemic frequently experience decreased physical strength and a higher risk of maternal morbidity and mortality. For instance, severe anemia increases the risk of death by 20% [6]. Anemic pregnant mothers have a higher risk of pre-term birth, fetal anemia, low birth weight (LBW), intrauterine fetal growth restriction, decreased gestational weight gain, and perinatal death [6,7,8]. Furthermore, anemia can result in a vicious cycle where a person’s ability to work is diminished, which can have an adverse economic effect on both the individual and society [9].

The WHO designed two broad strategies to address the issues of anemia and undernutrition: nutrition-sensitive and nutrition-specific. Nutrition-specific strategies address the proximal causes of anemia, primarily including inadequate dietary intake of hematopoietic nutrients such as iron or vitamin A, supplementation, and access to fortified foods. Dietary diversification is an approach designed to increase the availability, accessibility, and use of locally accessible and acceptable foods with a high micronutrient content and bioavailability annually [10]. Nutrition-sensitive strategies refer to interventions that address the underlying determinants of nutrition such as food security, health services, education, water, sanitation, and women’s empowerment rather than focusing solely on direct nutritional inputs. These strategies aim to create an enabling environment that supports improved nutrition outcomes. Examples include agricultural programs that promote the production and consumption of diverse and nutrient-rich foods, cash transfer programs that improve household food purchasing power, and women’s education and empowerment initiatives that enhance child care and feeding practices [11]. The Ethiopian government has implemented different strategies to prevent anemia and undernutrition among pregnant women, such as promoting dietary diversification and iron and folic acid supplementation, malaria prevention and treatment, utilization of bed nets, and deworming pills [12]. Also, the government has exerted considerable efforts to decrease maternal anemia and undernutrition by adopting and developing a nutrition and food strategy, program, and policy. Furthermore, the government developed the “Seqota Declaration” to guarantee year-round 100% access to sufficient food in the country by 2030 [13]. Moreover, health extension programs explicitly focusing on community-wide nutrition education are considered the most significant measures that can help promote adequate nutrition in the first 1000 days [12].

Despite all national commitments, measures, and efforts, anemia and undernutrition prevalence among pregnant women was high in the country as a whole, particularly in rural areas [5,14,15,16]. Also, there were considerable regional and urban/rural disparities in the prevalence of anemia and undernutrition at the national level [5], implying the need for further research into the landscape of anemia and undernutrition prevalence patterns in local settings. However, previous studies on the prevalence of anemia and undernutrition among pregnant women in Ethiopia focused primarily on individual-level factors, with little consideration given to community-level, household-level, and contextual factors. Furthermore, the factors contributing to anemia and undernutrition among pregnant women vary by region in Ethiopia; the prevalence of anemia and undernutrition is highly variable, and existing evidence is insufficient to develop comprehensive prevention strategies.

Undernutrition and anemia are two crucial indicators of maternal undernutrition that can impact the health of both the mother and the fetus. Maternal anemia and undernutrition are linked to an increased risk of pre-term birth, stillbirth, LBW, and neonatal deaths. If anemia and undernutrition coexist simultaneously, the risks increase [17]. Several studies have been conducted to assess anemia [18,19,20,21] and undernutrition [22,23,24,25,26,27,28] separately. However, to our knowledge, there is no evidence on the coexistence of anemia and undernutrition among pregnant women in developing countries, including Ethiopia, the effect of their coexistence on pregnancy outcomes, or whether specific interventions should be targeted toward women who share this dual burden. Therefore, we aimed to assess the prevalence and determinants of coexisting anemia and undernutrition among pregnant women in the Hawela Lida district of Sidama, Ethiopia.

The result of this study helps inform program managers, policymakers, decision-makers, and implementers about designing effective and efficient prevention strategies to improve maternal nutrition status and achieve the Sustainable Development Goals (SDGs). Furthermore, this study generated results that can inform maternal health champions by providing information on the determinants for coexisting anemia and undernutrition in the Sidama region.

## 2. Materials and Methods

### 2.1. Study Area

This study was done in the Hawela Lida district of the Sidama region, Ethiopia. Hawela Lida district is one of the 10 districts in the north zone of the Sidama region. It is located 289 km away from Addis Ababa, Ethiopia’s capital city. It consisted of 11 rural and two rural kebeles, representing the least administrative structure in Ethiopia.

### 2.2. Study Design, Period, Population and Sample Size

A community-based cross-sectional study was done among 515 randomly selected pregnant women from 1 to 25 June 2024. The study population consisted of randomly selected pregnant women in their first trimester who had resided in the district for at least six months prior to the survey. Pregnant women with severe illnesses, including but not limited to active tuberculosis, HIV/AIDS under antiretroviral therapy, chronic kidney disease, and any condition requiring hospitalization during the data collection period, were excluded from the study.

We calculated the minimum required sample size using OpenEpi version 3, assuming an anticipated anemia prevalence of 19.3% based on prior literature [14], with a 5% margin of error, 95% confidence level, and a design effect (DEFF) of 2 to account for the multi-stage sampling design. Given that previous studies did not report intraclass correlation coefficients (ICCs), we used a standard ICC value of 0.01 based on recommended estimates for community-based surveys [29]. The design effect (DEFF) was calculated using the formula DEFF = 1 + (m − 1) × ICC, where m is the average cluster size, and ICC is the intraclass correlation coefficient. The number of required clusters was approximated by multiplying the initial sample size by the ICC value. This yielded a minimum of approximately 2–3 clusters. However, to enhance statistical power and ensure variability between clusters, we included 10 kebeles (clusters) in the sampling frame. An additional 10% was added to the initial sample size to account for non-response. The final estimated sample size was 528 participants.

We used a multi-stage sampling method to recruit the study participants. The primary sampling unit was the district, and the Hawela Lida district was chosen purposefully from the Sidama region using the purposive sampling method. We purposively selected the Hawela Lida district because, immediately following this baseline study, an interventional study was implemented to investigate the effect of amaranth grain flatbread on anemia over six months. This district will provide geographic access to carefully implement, coordinate, supervise, and correct any unexpected problems near Hawassa City. The secondary sampling unit was kebeles, and 10 were selected using a simple random sampling procedure. The third sampling unit was households containing pregnant women, identified by conducting a house-to-house census in the selected kebeles. Pregnant women who were unavailable after three consecutive visits were considered non-respondents for this study. One mother was included by using a lottery sampling procedure when two or more pregnant mothers were in the chosen households.

### 2.3. Study Variables

The outcome variable was coexisting anemia and undernutrition. In this study, we assessed co-existing anemia and undernutrition (CAU) among pregnant women as a composite outcome, where anemia was defined by hemoglobin concentration <11 g/dL (adjusted for altitude, according to WHO guidelines), and undernutrition was defined by a mid-upper arm circumference (MUAC) <23 cm. The 23 cm MUAC cutoff was selected based on both international recommendations and national guidelines. According to the WHO and United Nations agencies, a MUAC measurement of <23 cm in pregnant women is widely used as a practical and reliable indicator of maternal undernutrition, especially in low-resource settings [1,2]. Importantly, the Ethiopian Federal Ministry of Health (FMoH) also recommends using a MUAC threshold of 23 cm to identify undernourished pregnant women who are at increased risk of adverse maternal and birth outcomes [30,31]. This cutoff is consistently applied in Ethiopia’s Community-Based Nutrition (CBN) programs and Maternal and Child Health (MCH) services, as outlined in the national nutrition guidelines [32]. In pregnancy, anemia is defined as hemoglobin <11 g/dL and is classified into three categories based on WHO criteria: mild (10.0–10.9 g/dL), moderate (7.0–9.9 g/dL), and severe (less than 7.0 g/dL) [3].

The independent variables are socioeconomic and demographic, such as women’s age, education status, husband’s education and occupation status, wealth index, family size, and mass media utilization; reproductive characteristics like planned pregnancy, previous history of stillbirth, women’s age at first marriage, and previous history of stillbirth; knowledge and practice of dietary diversity; and household food security. Every detail of individual and community-level variable measurements is provided in Supplementary File S1.

Training on model families refers to a structured, community-based intervention implemented as part of Ethiopia’s Health Extension Program. It involves the selection and education of “model families” through a minimum of 96 h of comprehensive training on essential health practices, including nutrition, hygiene, antenatal care, immunization, and family planning. Upon successful completion, these families are expected to implement the learned behaviors within their households and act as role models for neighboring community members. The initiative is designed to facilitate peer-to-peer learning and foster sustainable behavior change, ultimately aiming to improve health outcomes at the community level through practical demonstration and local ownership [33,34,35].

Nutrition knowledge among pregnant women was evaluated using a structured questionnaire adapted from previously validated tools used in similar low-resource settings. The tool included key questions related to food groups, nutrient-rich foods during pregnancy, consequences of poor nutrition, and recommended dietary practices. Each correct response was scored as 1 and incorrect or “don’t know” responses as 0. The total score was calculated by summing the individual item scores, and a composite score was generated. Based on the distribution of scores, we categorized nutrition knowledge into “good” and “poor” using the median value as a cut-off point. This approach is commonly used in population-based studies and allows for meaningful comparisons across groups [36,37].

### 2.4. Data Collection Tools and Techniques

The study tool was a standardized and pre-tested interviewer-administered questionnaire derived from similar previous research [38,39,40]. The questionnaire was initially created in English (see File S2). This tool was translated into Sidaamu Afoo (the native language used by the locals) and then converted back to English to ensure compatibility between the two versions. A linguistic expert in English and Sidaamu Afoo translated. The principal investigator (PI) and another individual with expertise in both languages reviewed the translated study tool. At that point, the inconsistency between the two language versions was corrected based on the identified issues. Twenty data collectors and four supervisors oversaw the data-gathering technique. The PI monitored and supervised the whole data-gathering process, correcting any issues.

### 2.5. Blood Collection and Serum Preparation Procedures

Standard procedures were followed for blood sample collection and laboratory analyses. Phlebotomists prepared the necessary materials (alcohol swabs, sterile gloves, a tourniquet, a vacutainer needle, a vacutainer tube, and a syringe) and collected blood from each participant. They were to clean and dry the collection site, apply a tourniquet, locate the vein, and prepare the needle and tube. Participants were instructed to make a fist, and the phlebotomist inserted the needle to collect 5 mL of blood. Afterward, the needle was withdrawn, pressure was applied to the puncture site, and it was safely discarded. The phlebotomist expressed gratitude to the participants for their cooperation.

Laboratory technologists performed hemoglobin analyses in all health posts. Each pregnant woman’s hemoglobin concentration was determined by taking a finger-prick blood sample with a HemoCue Hb 301 (HemoCue AB, Angelholm, Sweden). The site was disinfected, and a prick was conducted on the tip of the middle finger. The device used to measure hemoglobin concentration will eliminate the first drop of blood and collect the second drop to fill the micro cuvette. The cuvette holder held the microcuvette. We examined the meter’s performance daily using control standards to improve test reliability. While the meter was prepared to utilize capillary blood, the micro cuvette owner was first pulled to the loading position, and then the sample was filled continuously for the examination. Within 10 min of filling, it was placed in the holder and pushed into its measuring position. Finally, using WHO’s field survey recommendation techniques, the result was recorded and displayed after 15–60 s [3,41]. Before the data was entered, the hemoglobin level was corrected for altitude using the formula [3]. Serum extraction was performed within 45 min of sample collection at the nearby health institution to prevent hemolysis and contamination. The extracted serum samples were frozen at −20 °C and transported on dry ice to the Hawassa Referral Comprehensive Specialized Hospital for further analysis. Serum ferritin and C-reactive protein (CRP) were tested further in all samples with hemoglobin levels less than 11 g/dL. The Cobas 6000 e601 module from Roche (Berlin, Germany) was used to analyze serum ferritin, and the Cobas e501 module was used to analyze CRP. All laboratory procedures were carried out following standard operating procedures.

### 2.6. Data Quality Assurance

The PI trained the data collectors, field assistants, and field supervisors on the study tool for two days, emphasizing the significance of the research, the data collection procedure, objectives, sampling procedures, blood sample collection procedures, and ethical considerations. The data was collected using a well-designed, standardized, pre-tested, structured, face-to-face interviewer-administered questionnaire Following the pre-test, necessary adjustments were made before beginning the primary data collection procedure on the tool. The data collection procedure was strictly monitored. Data’s completeness, consistency, and accuracy were reviewed daily during data collection. The data was cleaned, coded, and exported to Stata 17 for further processing and analysis. Data collectors, field assistants, and supervisors were blinded to the exposure and outcome variables to reduce the likelihood of reporting bias. Furthermore, maximum efforts were made to reduce the bias risk by carefully selecting subjects representing the source population, increasing responses, and training data collectors, field assistants, and supervisors.

### 2.7. Ethics Statement

Ethical approval was obtained from the Institutional Review Board (IRB) of Hawassa University College of Medicine and Health Sciences (Ref: IRB/027/16). Written informed consent was secured from all participants after explaining the study purpose. Letters of support were received from the School of Public Health, Sidama Regional Health Bureau, Hawela Lida district, and local kebele leaders. Participants identified with severe anemia or undernutrition were referred to nearby health facilities for further care.

### 2.8. Data Analysis Techniques

Descriptive statistics (frequencies, percentages, means, and standard deviations) were used to summarize key variables. The household wealth index was computed using principal component analysis (PCA) [42].

A multi-level mixed-effects modified Poisson regression model with robust standard errors was used to identify individual- and community-level determinants of co-occurring anemia and undernutrition (CAU), accounting for the hierarchical structure of the data [43,44]. Four models were constructed: (1) null model (intercept-only), (2) model with individual-level variables, (3) model with community-level variables, and (4) full model including both levels. Variables with *p*-values < 0.25 in bivariable analysis and supported by literature were included in the multivariable model [45]. The intra-class correlation coefficient (ICC) and median prevalence ratio (MPR) were used to assess clustering effects [46]. Adjusted prevalence ratios (APR) with 95% confidence intervals were reported, and statistical significance was set at *p* < 0.05. Multicollinearity and effect modification were assessed; detailed analysis procedures and formulas are provided in Supplementary File S1.

## 3. Results

### 3.1. Socio-Demographic Characteristics of Research Subjects

We properly interviewed 515 pregnant women out of 528, resulting in a response rate of 97.53% for this study. The study participants’ mean (±SD) age was 25.89 (±4.53) years. Almost all 486 (94.4%) study participants were of Sidama ethnicity. Most research participants, 438 (85.2%), identified as Protestant Christians. Nearly all pregnant women were married, with 511 (99.2%) and 468 (90.9%) housewives, respectively.

### 3.2. Study Participants’ Reproductive Health Characteristics

The study participants’ mean age at first marriage (±SD) was 21.01 ± 3.10 years. A total of 81 (15.7%) women have previously had an abortion, and 93 (18.1%) have experienced an infection during their present pregnancy. Similarly, 49 (9.5%) and 28 (5.4%) women have had a history of stillbirths and neonatal deaths, respectively.

### 3.3. Prevalence of Coexisting Anemia and Undernutrition

**Figure 1:**
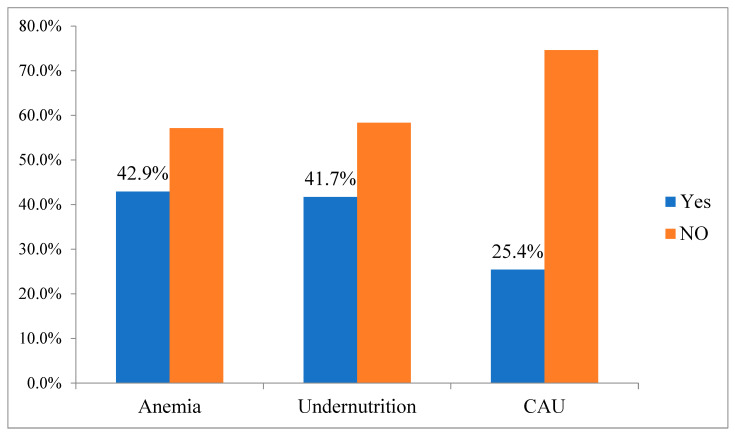
Prevalence of anemia, undernutrition and coexisting anemia, and undernutrition among pregnant women in the Hawela Lida district of the Sidama region, Ethiopia, 2024.

**Figure 2:**
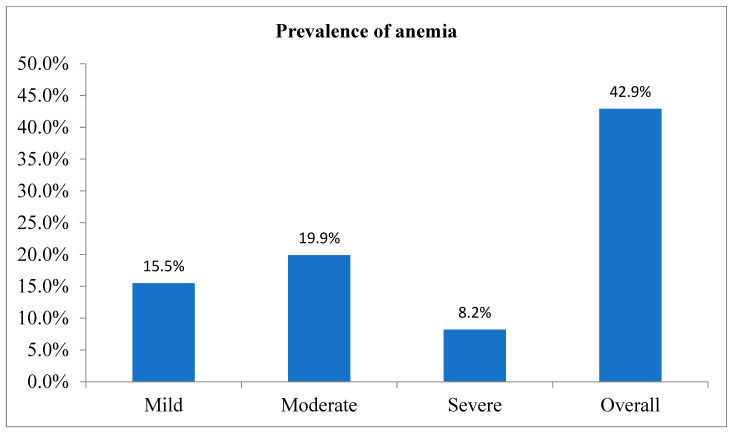
Prevalence of anemia among pregnant women in the Hawela Lida district of the Sidama region, Ethiopia, 2024.

The overall prevalence of CAU was 25.4% (95% CI: 21.9–28.9) (Figure 1). The prevalence of anemia among pregnant women was 42.9% (95% CI: 38.4–47.6), while undernutrition was 41.70% (95% CI: 37.3–45.6). Similarly, 15.5, 19.2, and 8.2% of study participants had mild, moderate, and severe anemia, respectively (Figure 2).

### 3.4. Determinants of Coexisting Anemia and Undernutrition

Pregnant women who had obtained model family training from health extension workers had a 34% higher likelihood of CAU than their counterparts (APR = 0.66; 95% CI: 0.45–0.96). Compared to women with adequate dietary diversity, pregnant women’s inadequate dietary diversity increased the likelihood of CAU prevalence (APR = 2.17; 95% CI: 1.43–3.28). Pregnant women in a food-insecure household had 51% more CAU prevalence than those in a food-secure household (APR = 1.51; 95% CI: 1.18–1.95). Women with poor nutrition knowledge had a higher prevalence of CAU than their counterparts (APR = 1.55; 95% CI: 1.06–2.26). While community-level road access decreased the likelihood of CAU prevalence (APR = 0.65; 95% CI: 0.43–0.98) as compared to community-level road inaccessibility, the likelihood of CAU prevalence was 6.19 times higher for pregnant women who lived in areas with high community-level women’s autonomy (APR = 6.19; 95% CI: 3.42–11.22) as compared to women who lived in areas with low community-level women’s autonomy (Table 1).

### 3.5. Random Effect Model and Model Fitness Information on CAU Prevalence

Our study revealed that the multi-level modified Poisson regression model fit better than the standard Poisson regression model (*p* < 0.001). According to the ICC value, involvement with kebeles explained 27.23% of the disparity in the prevalence of CAU among pregnant women. When two participants were randomly picked from different residential areas, the MPR value revealed that residual heterogeneity between the areas was related to 2.20 times the individual probability of having CAU. The final model revealed that the heterogeneity in CAU prevalence between residential areas remained statistically significant even after accounting for all potential contributing factors.

The CAU model fitness evaluation test showed that the empty model (AIC = 590.21, BIC = 598.70, and log-likelihood = −293.10) was the least fit. However, the models’ fitness increased dramatically, particularly that of the final model (AIC = 474.30, BIC = 512.50, and log-likelihood = −228.15). As a result, compared to the previous models, the final model fits the data well (Table 2).

## 4. Discussion

The prevalence of CAU among pregnant women was 25.4%. Inadequate dietary diversity, poor knowledge of nutrition, food-insecure status, training on model families, community-level women’s autonomy, and road inaccessibility were determinants of CAU among pregnant women.

The prevalence of CAU among pregnant women was 25.4%. There has been no previous publication on the prevalence of CAU and its determinants in Ethiopia or other African nations, limiting the comparability of our findings.

The significant association between pregnant mothers’ inadequate dietary diversity and CAU prevalence may be explained by the fact that women who consume a diverse diet obtain a wider range of nutrients, making their overall nutrient intake higher than mothers with lower dietary diversity scores. Furthermore, this could be because women do not consume extra meals during pregnancy, and maternal dietary habits, socio-cultural beliefs, and food taboos can all affect nutrition during pregnancy. Preventing malnutrition in all forms, before and during pregnancy, depends on appropriate nutrition practices, essential nutrition services, and healthy diets. Thus, all prenatal care must equip pregnant women with dietary teaching and counseling, which should be expanded. Furthermore, the researchers contended that this could be expressed as follows: eating a diverse diet is vital for obtaining all the nutrients required to prevent undernutrition caused by nutritional deficiencies [47]. Enhancing nutrition education for pregnant women particularly through community health platforms such as health extension workers is crucial to improving dietary diversity and the consumption of iron-rich foods.

This research found that the training on model families decreased the prevalence of CAU. The possible rationale is as follows: women who have received model family training from health extension workers tend to have good knowledge of nutrition during pregnancy and lactation or nutrition benefits during the first 1000 days, a positive attitude toward nutritious diets, and are able to avoid food taboos culture, possess good health-seeking behavior, and obtained right information on the benefits of dietary diversity practices. Expanding model family training programs, which were found to be associated with reduced co-occurrence of anemia and undernutrition, can empower households with essential health and nutrition knowledge, promoting sustainable behavior changes at the community level.

The positive relationship between household food insecurity and CAU prevalence may be because a lack of food in the household leads to an insufficient daily nutrient intake, resulting in undernutrition in pregnant women. Researchers also claimed that one of the primary underlying causes of undernutrition is household food insecurity, which occurs when a household does not always have physical, social, or financial access to enough food to meet its nutritional needs for a healthy life. Women tend to consume less than men, which could be attributed to limited food availability. Furthermore, the women used coping methods to reduce their food intake while nourishing their small children and newborns during a food shortage. As a result, improving community food security for homes is critical to avoiding and eliminating acute undernutrition and its negative long-term consequences [48]. Promoting household food security through social protection measures and income-generating interventions is essential for sustaining maternal nutrition and reducing the risk of undernutrition during pregnancy.

Poor nutritional knowledge among pregnant women was associated with an increased prevalence of CAU, possibly because a limited understanding of nutrition often leads to inadequate dietary intake and, consequently, undernutrition. Furthermore, women with nutrition knowledge may better perceive the benefits of eating a healthy and appropriate diet during their pregnancy and be prepared to adopt it.

This study documented that high community-level women’s autonomy decreased the prevalence of CAU. Communities with significant women’s autonomy had higher knowledge about nutrition and health-seeking behaviors. Autonomous mothers are more educated and financially independent, have more job opportunities, and understand the importance of adequate dietary diversity. Another factor might be that literate populations utilize more community-level mass media, which may increase community discussion about maternal health issues. According to the WHO report, mothers who reside in affluent communities may have been exposed to more mass media, which has increased their awareness and knowledge of nutrition and appropriate dietary diversification practices [49]. Research results in low-income nations further support this idea [50,51].

The prevalence of CAU increased for pregnant women who lived in poor, road-accessible communities. One possible rationale is that community-level road inaccessibility reduces access to fundamental services such as health and education. Researchers also highlighted Ethiopia’s geographic differences in health services [52,53,54]. Another reason might be that healthcare providers give less attention to screening and linking undernutrition pregnant women from road-inaccessible places, which contributed to the high prevalence of CAU in settings with low road accessibility. Improving access to health and nutrition services in underserved areas by addressing infrastructural barriers such as poor road accessibility is essential for ensuring timely care and support for pregnant women at risk of anemia and undernutrition.

### Limitations of the Study

Seasonality was not considered as a confounder in our analysis due to the cross-sectional design of the study, which captured data at a single point in time. We recognize that seasonal variations in food availability and dietary intake could influence maternal nutritional status. Thus, future longitudinal studies are recommended to better assess seasonal effects on maternal nutrition. In this study, we assessed anemia using hemoglobin concentration, which is a standard indicator that is widely recommended by the WHO for defining and classifying anemia, particularly in large-scale surveys and resource-limited settings [3]. Other anemia-related parameters such as hematocrit, serum ferritin, or transferrin saturation would indeed offer a more comprehensive understanding of anemia etiology. However, due to logistical and financial constraints, and the limited laboratory infrastructure in our study area, we were unable to collect these additional biomarkers. Portable hemoglobinometers were used as they are practical and validated for community-based anemia screening.

## 5. Conclusions

One in four pregnant women had CAU in the study setting. Inadequate dietary diversity, poor knowledge of nutrition, food-insecure status, training on model families, community-level women’s autonomy, and road inaccessibility were determinants of CAU among pregnant women. Thus, any programs related to maternal nutritional improvement strategies should address these determinants to decrease the high prevalence of CAU. Likewise, intervention approaches in particular should be considered for pregnant women with poor knowledge of nutrition, pregnant women who have not obtained training in a model family, and pregnant mothers who have inadequate dietary diversity. Moreover, there is a significant level of food insecurity in the research area, which adds to the high rate of CAU among pregnant women. Designing and enhancing the promotion of food security policies based on Ethiopian national directives and WHO guidelines is a thoughtful demand. Furthermore, there is an urgent need for nutritional screening for women living in poor, road-accessible areas to circumvent the high prevalence of CAU. Finally, reforms for women’s autonomy, raising communities with low women’s autonomy with the help of rural health extension workers, must be considered.

## Figures and Tables

**Table 1:** The determinants of coexisting anemia and undernutrition (CAU) among pregnant women in the Hawela Lida district of the Sidama region, Ethiopia, 2024.

Variables	Coexisting Anemia and Undernutrition		CPR (95% CI)	APR (95% CI)
	Yes	No		
Individual-level determinants				
Women’s education				
Have formal education	80 (23.2)	265 (76.8)	Ref	Ref
No formal education	51 (30.0)	119 (70.0)	1.14 (0.85, 1.54)	0.87 (0.75, 1.01)
Family size				
Small	97 (23.9)	309 (76.1)	Ref	Ref
Large	34 (31.2)	75 (68.8)	1.12 (0.83, 1.50)	0.92 (0.64, 1.31)
Women’s occupation status				
Housewife	113 (24.4)	350 (75.4)	Ref	
Merchant	11 (36.7)	19 (63.3)	0.99 (0.55, 1.77)	0.98 (0.59, 1.62)
Government employee	7 (31.8)	15 (68.2)	0.73 (0.19, 2.78)	0.78 (0.31, 1.95)
Decision-making power of women				
Autonomous	66 (20.9)	250 (79.1)	Ref	Ref
Non-autonomous	65 (32.7)	134 (67.3)	2.26 (0.89, 5.78)	1.37 (0.41, 4.54)
Model family training				
Not obtained	45 (29.8)	106 (70.2)	Ref	Ref
Obtained	86 (23.6)	278 (76.4)	0.63 (0.37, 1.06)	0.66 (0.45, 0.96) *
Food security status				
Secure households	46 (14.4)	274 (85.6)	Ref	Ref
Insecure households	85 (43.6)	110 (56.4)	2.89 (1.50, 5.57)	2.17 (1.43, 3.28) **
Dietary diversity status				
Adequate	39 (15.9)	206 (84.1)	Ref	Ref
Inadequate	92 (34.1)	178 (65.9)	1.91 (1.50, 2.42)	1.51 (1.18, 1.95) **
Women’s knowledge about nutrition				
Good	36 (13.6)	228 (86.4)	Ref	Ref
Poor	95 (37.8)	156 (62.2)	2.40 (1.59, 3.61)	1.55 (1.06, 2.26) *
Food security status				
Secure households	46 (14.4)	274 (85.6)	Ref	Ref
Insecure households	85 (43.6)	110 (56.4)	2.89 (1.50, 5.57)	2.17 (1.43, 3.28) **
Dietary diversity status				
Adequate	39 (15.9)	206 (84.1)	Ref	Ref
Inadequate	92 (34.1)	178 (65.9)	1.91 (1.50, 2.42)	1.51 (1.18, 1.95) **
Women’s knowledge about nutrition				
Good	36 (13.6)	228 (86.4)	Ref	Ref
Poor	95 (37.8)	156 (62.2)	2.40 (1.59, 3.61)	1.55 (1.06, 2.26) *
Women’s attitude towards nutrition				
Positive	92 (26.7)	252 (73.3)	Ref	Ref
Negative	39 (22.8)	132 (77.2)	0.87 (0.50, 1.52)	0.78 (0.30, 2.01)
Community-level determinants				
Place of residence				
Urban	10 (11.0)	81 (89.0)	Ref	Ref
Rural	121 (28.5)	303 (71.5)	3.13 (0.36, 26.95)	3.53 (0.33, 37.40)
Community-level wealth status				
High	50 (16.4)	255 (83.6)	Ref	Ref
Low	81 (38.6)	129 (61.4)	1. 68 (0.67, 4.17)	1.25 (0.76, 2.06)
Community-level distance				
Not big problem	77 (23.6)	249 (76.4)	Ref	Ref
Big problem	54 (28.6)	135 (71.4)	0.96 (0.45, 2.06)	0.78 (0.30, 2.02)
Community-level literacy				
High	13 (8.8)	134 (91.2)	Ref	Ref
Low	118 (32.1)	250 (67.9)	4.28 (0.83, 21.98)	2.16 (0.49, 9.54)
Community-level road access				
Inaccessible	18 (36.0)	32 (64.0)	Ref	Ref
Accessible	113 (24.3)	352 (75.7)	0.73 (0.45, 1.17)	0.65 (0.43, 0.98) *
Community-level autonomy				
High	16 (8.2)	178 (91.8)	Ref	Ref
Low	115 (35.8)	206 (64.2)	7.75 (3.52, 17.05)	6.19 (3.42,11.22) **

APR: adjusted prevalence ratio; *: significant association (*p* < 0.05); **: highly significant association (*p* < 0.01); CI: confidence interval; CPR: crude prevalence ratio; Ref: reference group.

**Table 2:** Multi-level modified Poisson regression analysis result of model selection criteria and random effect model information.

Measure of Variation	Model 0 (95% CI)	Model 1 (95% CI)	Model 2 (95% CI)	Model 3 (95% CI)
Variance of intercept	0.69 (0.16, 3.02)	0.58 (0.08, 3.92)	0.57 (0.20, 1.58)	0.49 (0.14, 1.73)
ICC percentage	27.23 (10.49–54.40)			
MPR	2.20 (1.46–5.21)			1.95 (1.42, 3.49)
Model fitness				
Log-likelihood ratio	−293.10	−257.72	−245.67	−228.15
AIC	590.21	533.45	505.35	474.30
BIC	598.70	571.64	535.06	512.50

MPR: Median prevalence ratio; ICC: Intra-class correlation coefficient; AIC: Akaike information criteria; BIC: Bayesian information criteria; CI: confidence interval.

## Data Availability

Data is available in the Supplementary File S3.

## Supplementary Materials

The following supporting information can be downloaded at https://www.mdpi.com/article/10.3390/healthcare13131495/s1, Table S1: Description of study variables; Table S2: Some of the variables and given values used to facilitate the computation of wealth index; File S1: Detailed Data Analysis Procedures and Formulas; File S2: English version of study questionnaire; File S3: Data set. References [55,56,57,58,59,60,61,62,63,64,65,66,67,68,69,70,71,72,73,74] are cited in the supplementary materials.

## Abbreviations

The following abbreviations are used in this manuscript:

AIC	Akaike information criteria
APR	Adjusted prevalence ratio;
BIC	Bayesian information criteria
CI	Confidence interval;
CPR	Crude prevalence ratio
EDHS	Ethiopian Demographic and Health Survey
FANTA	Food and nutrition technical assistance
FAO	Food and agriculture organization
HCPs	Health Care Providers
HEW	Health extension worker
ICC	Intra-class correlation coefficient
IRB	Institutional review board
IUGR	Intrauterine growth retardation
MPR	Median prevalence ratio
NGO	Non-governmental organization
PI	Principal Investigator
SD	Standard deviation
VIF	Variance inflation factor
WHO	World Health Organization
WRA	Women of reproductive age
