# Supplementary material for: The Determinants of Coexisting Anemia and Undernutrition Among Pregnant Women in Southern Ethiopia: A Multi-Level Analysis
Source: Healthcare (Basel). 2025 Jun 23;13(13):1495. doi: 10.3390/healthcare13131495 (PMC12249147; doi:10.3390/healthcare13131495)
Supplement: Supplementary file 1 [file healthcare-13-01495-s001.zip › S1 File.pdf]

S1 File Table S1: Description of study variables

**Individual-level variables**	
Distance from the health facility (HF)	The distance from the HF is considered as close to the HF if a woman was reported to travel less than 5 km or have walking hours less than 30 by foot to reach the nearest HF[55,56].
Use of mass media	The use of mass media was generated by combining whether a study participant listens to the radio, watches television, and reads the newspaper and categorized as "yes" if the respondent is exposed to at least 1 of the 3 media types and "no" otherwise[57,58].
Family size	Family size is defined as the total number of individuals existing in the household and was categorized as small when it was < 5 and large (≥ 5)[59].
Women's knowledge regarding nutrition	The women's knowledge regarding nutrition was measured using the 16 knowledge questions. The correct answer was assigned a score of 1, while an incorrect answer was assigned a score of 0. Hence, the total expected knowledge score ranged from 0 to 16. Hence, an increased total sum of the score was considered to indicate increased knowledge of nutrition on the scale. Finally, it was classified into good and poor using the mean value. Pregnant women who scored below the mean were considered to have poor knowledge.
Women's attitudes regarding nutrition	The women's attitudes regarding nutrition were assessed using the 15 attitude questions. Each attitude question was measured using a 5-point Likert scale from strongly disagree (1) to strongly agree (5). The total expected

	attitude score ranged from 15 to 75. Then, the value of each item scored on the Likert scale by respondents was summed, and the values were considered as discrete scale data. Hence, an increased total sum of the score was considered to indicate an increased positive attitude on the scale. Finally, they were classified into positive and negative using the mean value. Pregnant women who scored below the mean were considered to have a negative attitude.
Formal education	Formal education is education extending from primary to secondary or higher education and requires an organized and careful purpose that concretizes itself in an official curriculum that is applied with a defined calendar and timetable[60]
Women's autonomy	A woman was considered autonomous if the woman could decide when and where to use MHS or decide on healthcare spending by herself alone or with her husband together and non-autonomous otherwise. The determination was made using a woman's self-report [61]
Dietary diversity scores	The dietary diversity scores were calculated using Food and Agriculture Organization (FAO) guidelines[62] After computation, the score was divided into two groups, namely adequate and inadequate. Nine food groups were created from all of the pregnant women's reported foods and drinks consumed the day before the survey: cereals and starchy staples; oils and fats; dark green leafy vegetables and vitamin A-rich fruits and vegetables; legumes; nuts and seeds; other fruits and vegetables; meat and fish; organ meat; milk and milk products; and

	eggs. Pregnant women who have eaten a food in each subgroup (at least once) received a score of 1, and otherwise, a 0 was assigned.
The household food insecurity questions	The household food insecurity questions were based on food and nutritional technical assistance (FANTA) version 3 and were modified for the local context; they contained 27 questions[63] The first nine questions were answered "yes" or "no," and the results were divided into four groups: food secure, mildly food insecure, moderately food insecure, and severely food insecure.
**Community-level variables**	
Place of residence	The place of residence was categorized as urban or rural.
Community-level women's literacy	The aggregate value of community-level women's literacy was generated by the percentage of the population of women in the cluster that had at least a primary level of literacy derived from the individual participants' data. It was categorized as a "high" concentration of literate women in the *kebeles* if the percentage of women who had at least primary-level education was ≥50% and "low" otherwise[64, 65]
Community-level poverty	The aggregate value of community-level poverty was generated by the percentage of households in the cluster in the poorest and poorer quintile derived from the individual participants' data. It was categorized as a "high" concentration of poverty in the *kebeles* if the percentage of households in the poorest and poorer quintile was ≥50% and "low" otherwise[66, 67]

Community-level social media use	The aggregate value of community-level social media use was generated by the percentage of study participants who listen to the radio, watch television, and read the newspaper in clusters derived from the individual participants' data. It was categorized as a **"**high" concentration of social media use in the *kebeles* if the proportion of study participants who use at least one social media was ≥50% and "low" otherwise[68,69]
Distance from nearest HF	The distance from the nearest HF was considered as "close" to the HF if a woman reported a walking hour of less than 30 minutes by foot to reach the nearest HFs and "far" otherwise[55] The aggregate value of community-level distance was generated by the percentage of study participants with walking hours to the nearest HF of less than 30 minutes in a cluster derived from the individual participants' data. It was categorized as **"**not big problem" in the *kebeles* if ≥50% of the study participants were reported as "close" and "a big problem" otherwise [68, 69]

The wealth index was calculated by using principal component analysis (PCA) as a combined indicator of life standard based on 42 questions related to the ownership of prudently selected household assets like the owner of the house, materials used for house construction, number of rooms in a house, size of agricultural land, presence of a herd or farm animals and livestock, types of fuel used for cooking, and possession of improved sanitation and a water facility[70,71]. The multiple response variables were categorized into binary responses (yes/no) and "I don't know" responses were often coded as 999 to 0 (Table 3). Similarly, the "I don't know" response and any missing values were often coded as 999 to 0 for the continuous variables[70] The predictors that can differentiate

between comparatively "poor" and "rich" households were selected using a simple frequency analysis. Thus, our PCA did not comprise any assets or variables that were possessed by less than 5% or more than 95% of the individuals in the sample[70,71] Finally, the component factors or wealth index scores were ranked into five classes such as lowest, second-lowest, middle, second-highest, and highest[71,74]. The PCA was carried out for the computation of the wealth index[72] All the basic assumptions of PCA were checked before ranking the components' factor scores into wealth quintiles. We removed the variables from the PCA that did not satisfy assumptions such as a Kaiser–Meyer–Olkin (KMO) measure of sampling adequacy of less than 0.5, commonalities less than 0.5, and variables that contain a complex structure (high loading correlation ≥0.4 on greater than one component)[73,74]

S1 File Table S2: Some of the variables and given values used to facilitate the computation of wealth index.

S.no	Variables	Given values
1	Main source of drinking water	Improved: Piped water, tube well or borehole, protected well, and protected spring = 1<br>Unimproved: Unprotected well, unprotected spring, and lake/pond/stream/canal = 0
2	Main source of water used for other purposes such as cooking and hand washing	Improved: Piped water, tube well or borehole, protected well, and protected spring = 1<br>Unimproved: Unprotected well, unprotected spring, lake/pond/stream/canal, and surface water (river/dam) = 0
3	Where is that water source located?	In own dwelling or yard/plot = 1

		Elsewhere = 0
4	Type of toilet facilities	Improved: comprise any non-shared toilet of the subsequent kinds: pour/flush toilets to septic tanks, piped sewer systems, pit latrines; pit latrines with slabs; ventilated improved pit (VIP) latrines; and composting toilets = 1<br>Unimproved: Pit latrine without slab/open pit, bucket toilet, and hanging toilet = 0
5	Where is this toilet facility located?	In own dwelling or yard/plot = 1<br>Elsewhere = 0
6	Type of fuel the household mainly use for cooking	Clean fuels include electricity, liquefied petroleum gas (LPG), natural gas, and biogas = 1<br>Solid fuels include coal, charcoal, wood, straw/shrub/grass, agricultural crops, and animal dung = 0
7	Where is the cooking usually done?	In the house and outdoors = 0<br>In a separate building = 1
8	Who is the owner of the house?	Me = 1<br>Rental, family, and relative = 0
9	Main material of the roof of the house	Natural roofing (no roof, mud, and sod) = 0<br>Rudimentary and finished roofing = 1

10	Main material of the floor of the house	Natural floor (earth/sand, or dung) = 0<br>Rudimentary and finished floor = 1
11	Main material of the wall of the house	Natural walls (no walls, cane/palm/trunks/bamboo/ree, or dirt) = 0<br>Rudimentary and finished walls = 1
12	All other categorical variables were considered as a yes or no form	Yes = 1 and no =0
13	All continuous variables were treated as continuous	
14	“I don’t know” response often coded as 999 for categorical variables	999 = 0
15	“I don’t know” response and any missing value often coded as 999 to 0	999 and missing value = 0

Supplementary File S1. Detailed Data Analysis Procedures and Formulas

1. Data processing and variable preparation

- Quantitative variables were cleaned, recoded, and categorized prior to analysis.
- Nutrition knowledge was scored by assigning "1" for correct answers and "0" for incorrect or "I don't know" responses. Scores were summed to generate a composite score, then dichotomized using the median value as a cutoff (poor vs. good knowledge).
- Dietary diversity was calculated based on a 24-hour recall of food groups and categorized using FAO guidelines.
- Household food insecurity was assessed using the Household Food Insecurity Access Scale (HFIAS), and classified as food secure vs. insecure.
- Wealth index was generated via Principal Component Analysis (PCA) using household assets, housing conditions, and access to services.

2. Sample size design effect and ICC estimation

To account for clustering in kebeles, the design effect (DEFF) was calculated as follows:

DEFF=1+(m−1)×ICC\text{DEFF} = 1 + (m - 1) \times \text{ICC}

with the following variables:

- mm = average number of subjects per cluster.
- ICC\text{ICC} = intra-class correlation coefficient (assumed 0.01 based on WHO and methodological recommendations for cluster surveys in LMICs).

3. Model building approach

A multi-level (mixed-effects) modified Poisson regression was used to estimate adjusted prevalence ratios (APR). Four models were constructed:

- **Model 1**: Null/intercept-only model (to assess clustering effect).
- **Model 2**: Individual-level variables only.
- **Model 3**: Community-level variables only.
- **Model 4**: Full model with individual and community-level variables.

4. Clustering assessment

To evaluate clustering and between-kebele variance, the following equation was used:

Intra-class Correlation Coefficient (ICC): $\text{ICC} = \frac{\sigma^2_{u}}{\sigma^2_{u} + \pi^2/3}$

with the following variables:

- σ^2_{u} = variance between kebeles (random intercept).
- $\pi^2/3 \approx 3.29$ is the assumed level-1 variance for binary outcomes in logistic-type models.

Median prevalence ratio (MPR): $\text{MPR} = e^{0.95 \times \sqrt{\sigma^2_{u}}}$

with the following variables:

- σ^2_{u} is the estimated between-cluster variance.
- MPR quantifies unexplained heterogeneity between kebeles.

5. Variable selection for multivariable model

- Variables with $p < 0.25$ in bivariable analysis were considered for multivariable modeling.

- Additional inclusion was based on biological plausibility and previous literature.
- A backward elimination approach was applied, retaining variables with $p < 0.05$ in the final model.

6. Multicollinearity and effect modification

- **Multicollinearity** was assessed using the Variance Inflation Factor (VIF):

  VIF<5⇒no significant multicollinearity\text{VIF} < 5 \Rightarrow \text{no significant multicollinearity}

- **Effect modification** was examined by testing interactions between key variables (e.g., food insecurity × dietary diversity). Where significant ($p < 0.05$), stratified analysis was considered.

7. Reporting

- Effect estimates are presented as adjusted prevalence ratios (APRs) with 95% confidence intervals (CIs).
- Statistical significance was considered at $p < 0.05$.
- All analyses were conducted using Stata v17.0.

Effect modification result

We entered the interaction terms in the final model for women's education and women's knowledge of nutrition, dietary diversity, and food security status, community-level wealth status and community-level literacy rate, community-level distance to nearest health

facilities, and community-level road access to see if women's education modifies the effect of women's knowledge of nutrition and if food security modifies the effect of dietary diversity, etc. None of the interaction terms was statistically significant, implying the absence of a significant effect modification.
