# Supplementary material for: The Determinants of Coexisting Anemia and Undernutrition Among Pregnant Women in Southern Ethiopia: A Multi-Level Analysis
Source: Healthcare (Basel). 2025 Jun 23;13(13):1495. doi: 10.3390/healthcare13131495 (PMC12249147; doi:10.3390/healthcare13131495)
Supplement: Supplementary file 1 [file healthcare-13-01495-s001.zip › S2 File.pdf]

English version questionnaire

Hawassa University

School of Public Health

Questionnaire Designed to Study the Determinants of Coexisting Anemia and Undernutrition Among Pregnant Women in Southern Ethiopia: A Multilevel Analysis

PART I: General Information

01. Name of *kebeles*: ____________

02. House number ____________________

03. Individual ID __________________

04. Date of interview: ____________time interview started __________ ended at ____________

05. Respondent available on:

1. 1st visit.
2. 2nd visit.
3. 3rd visit.

Part I: Socio-demographic characteristics of study participants

Instruction: First, tell interviewee that you are going to ask her questions about herself. Second, properly explain to the study participant each question to elicit genuine response from the respondents. Then, request her to correctly respond to the questions. Please write the responses based on the respondent's genuine responses (in field to be filled by data collectors).

S.no	Questions	Response categories	Skip to	Remark
101	What is your age (in completed years)?	__________ years		
102	To which ethnic group do you belong?	1. Sidama<br>2. Amhara<br>3. Oromo<br>4. Gurage<br>5. Wolayita<br>88.Other, specify_____		
103	What is your religious affiliation?<br>(encircle the response)	1. Protestant Christian<br>2. Orthodox Christian<br>3. Catholic<br>4. Muslim<br>5.Other (specify) __________		
104	What is your highest educational level you have completed?	1. Cannot read and write<br>2. Can read and write only<br>3. Primary education (1-8)<br>4. Secondary education (9-12)<br>5. College diploma<br>6. College/university degree and		

		above		
105	What is your occupation?	1. Housewife<br>2. Farmer<br>3. Governmental employee<br>4. Merchant<br>5. Housemaid<br>6. Other (specify)………………		
106	What is your marital status?	1. Not ever married……………<br>2. Married<br>3. Divorced<br>4. Separated<br>5. Widowed<br>6. Cohabiting	Q110	
107	What is your husbands age in complete years?	________________		
108	What is your husband's occupation?	1. Governmental employee<br>2. Merchant<br>3. Farmer<br>4. Daily labourer<br>5. NGO employee<br>6. Private organization employee<br>7. Other (specify)………………		
109	What is your husband's educational status?	1. Cannot read and write<br>2. Can read and write only		

		3. Primary education (1-8)<br>4. Secondary education (9-12)<br>5. College diploma<br>6. College/university degree and above		
110	What is your family size? (In number)	________		
111	Which social media do you use?	1. Listens the radio<br>2. Watches the television<br>3. Reads the newspaper<br>4. All<br>5. Others		

Part II: Household wealth index of study participants

S.no	Questions	Response categories	Skip to	Remark
201	What is the main source of drinking water for members of your household?	1. Piped water<br>2. Tube well or Borehole<br>3. Protected well<br>4. Unprotected well<br>5. Protected spring<br>6. Unprotected spring<br>7. Rainwater<br>8. Surface water (River/dam)<br>9. Lake/pond/stream/canal<br>10. Bottled water		
202	What is the main source of water used by your household for other purposes such as cooking and handwashing?	1. Piped water<br>2. Tube well or Borehole<br>3. Protected well<br>4. Unprotected well<br>5. Protected spring<br>6. Unprotected spring<br>7. Rainwater<br>8. Surface water (River/dam)<br>9. Lake/pond/stream/canal<br>10. Bottled water		
203	Where is that water source located?	1. In own dwelling<br>2. In own yard/plot<br>3. Elsewhere		
204	How long does it take to go there, get water, and come back?	Minutes . . . .<br>998. Don't know		
205	In the past two weeks, was the water from this source not available for at least one full day?	1. Yes<br>0. No<br>998. Don't know		
206	Do you do anything to the water to make	1. Yes		

	it safer to drink?	0. No<br>998. Don't know		
207	What kind of toilet facility do members of your household usually use?<br><br>IF NOT POSSIBLE TO DETERMINE, ASK PERMISSION TO OBSERVE THE FACILITY.	1. Flush or pour flush toilet<br>2. Ventilated improved pit latrine<br>3. Pit latrine with slab<br>4. Pit latrine without slab/open pit<br>5. Compositing toilet<br>6. Bucket toilet<br>7. Hanging toilet<br>8. No facility/bush/field		
208	Do you share this toilet facility with other households?	1. Yes<br>0. No		
209	Including your own household, how many households use this toilet facility?	____________________<br>998. Don't know		
210	Where is this toilet facility located?	1. In own dwelling<br>2. In own yard/plot<br>3. Elsewhere		
211	What type of fuel does your household mainly use for cooking?	1.Electricity<br>2. Liquefied petroleum gas<br>3. Natural gas<br>4.Biogas<br>5.Keresone<br>6. Charcoal<br>7. Wood<br>8. Straw/shrubs/grass<br>9. Agricultural crop		

		10. Animal dung<br>11.No food cooked in household		
212	Is the cooking usually done in the house, in a separate building, or outdoors?	1. In the house<br>2. In a separate building<br>3. Outdoors<br>4. Others (specify)………………		
213	Do you have a separate room which is used as a kitchen?	1. Yes<br>0. No		
214	Who is the owner of the house?	1. Me<br>2. Rental<br>3. Family<br>4. Relative<br>5. Others (specify)…………...		
215	How many rooms in this household are used for sleeping?	-------------------------		
216	Main material of the roof of the house?	1. Natural roofing (no roof, mud, and sod)<br>2. Rudimentary roofing (rustic mat/plastic sheet, reed/bamboo, wood planks, and cardboard)<br>3. Finished roofing (metal/corrugated iron, wood, calamine/cement, ceramic tiles, roofing shingles)		
217	Main material of the floor of the house?	1. Natural floor (Earth/sand, dung)<br>2. Rudimentary floor (wood planks, and palm/bamboo)<br>3. Finished floor (parquet or polished wood, vinyl or asphalt		

		strips/ plastic tiles, cement, ceramic tiles, carpet)		
218	Main material of the floor of the house?	1. Natural walls (no walls, cane/palm/trunks/bamboo/reef, dirt)<br>2. Rudimentary walls (bamboo with mud, stone with mud, uncovered adobe, plywood, cardboard, and reused wood)<br>3. Finished floor (cement, stone with lime/cement, bricks, cement blocks, covered adobe, wood planks/shingles)		
219	Does this household own any livestock, herds, other farm animals, or poultry?	1.Yes<br>0.No	 222	
220	How many of the following animals does this household own?<br>IF NONE, RECORD ‘00’.<br>IF 95 OR MORE, RECORD ‘95’.<br>IF UNKNOWN, RECORD ‘98’.<br>a) Milk cows, oxen or bulls?<br>b) other cattle?<br>c) Horses/donkeys/mules?<br>d) Camels?<br>e) Goats?<br>f) Sheep?<br>g) Chickens/poultry?<br>h) Beehives?	a) Milk cows, oxen or bulls………<br>b) other cattle………………….<br>c) Horses/donkeys/mules……….<br>d) Camels……………………...<br>e) Goats………………………<br>f) Sheep………………………….<br>g) Chickens/poultry…………….<br>h) Beehives…………………….		

221	Do you have separate rooms for cattle?	1.Yes<br>0.No		
222	Does any member of this household own any agricultural land?	1.Yes<br>0. No	224	
223	How many hectares of agricultural land do members of this household own?	--------------------------hectares		
224	Does your household have:<br>a) Electricity?<br>b) A radio?<br>c) A television?<br>d) A non-mobile phone?<br>e) A computer?<br>f) A refrigerator?<br>g) A table?<br>h) A chair?<br>i) A bed with cotton/sponge/spring mattress?<br>j) An electric mitad?<br>k) A kerosene lamp/pressure lamp?	Yes No<br>a) Electricity…….1 0<br>b) Radio…………1 0<br>c) Television………1 0<br>d) Non-mobile phone.1 0<br>e) Computer…………1 0<br>f) Refrigerator………1 0<br>g) Table………………1 0<br>h) Chair………………1 0<br>i) Bed with cotton/sponge/spring mattress…………1 0<br>j) Electric mitad…….1 0<br>k) Kerosene lamp/pressure lamp………………1 0		
225	Does any member of this household own:<br>a) A watch?<br>b) A mobile phone?<br>c) A bicycle?<br>d) A motocycles/scooter?<br>e) An animal drawn cart?<br>f) A car/truck?	Yes No<br>a) Watch . . . . 1 0<br>b) Mobile phone . . .. 1 0<br>c) Bicycle. . . . . 1 0<br>d) Motocycles/scooter... 1 0<br>e) Animal drawn cart . . .1 0<br>f) Car/truck . . . . .1 0<br>g) Boat with motor 1 0		

	g) A boat with motor?<br><br>h) A bajaja?	h) Bajaja . . . . ………1 0		
226	Does any member of this household have a bank account?	1. Yes<br>0. No		
227	Does any member of this household have a microfinance account?	1. Yes<br>0. No		

Part III: Reproductive history of study participants

S.no	Questions	Response categories	Skip to	Remark
301	What was your age when you were first married?	________________________<br>998. I don't know		
302	What was your age at your first pregnancy?	________________________<br>998. I don't know		
303	Have you ever become a pregnant?	0. No<br>1. Yes	3	
304	If yes for question 303, how many times?	---------------------------		
305	Have you ever faced abortion?	0. No<br>1. Yes	307	
306	If yes for question 305, how many times?	----------------------------		
307	Have you ever delivered a child?	0. No<br>1. Yes	311	
308	If yes for question 306, how many times?	-----------------------------		
309	How many of them were live births?	------------------------------		
310	How many of them were delivered at home?	---------------------------		
311	Infection during the current pregnancy (e.g., UTI, periodontal disease)	0. No<br>1. Yes		
312	Have you ever experienced death of foetus or stillbirth (fatal death at or after	0. Yes<br>1. No		

	28 weeks of pregnancy)?	998. I don't remember		
313	Have you ever experienced death of neonate (age less than seven days)?	0. Yes<br>1. No<br>998. I don't remember	end	
314	If yes to Q 313, how many times?	_____________		
315	If yes to Q 313, where was the neonate born?	1. Home<br>2. Health institution<br>3. Other (specify) _________		

Part IV: Food groups used to calculate dietary diversity score

Questions	Food group	Food example	Answer
1	Cereals	Corn/maize, rice, wheat, sorghum, millet or any other grains or foods made from these (e.g. Bread, noodles, porridge or other grain products) + insert local foods e.g. Ugali, nshima, porridge or paste	Yes = 1<br>No = 0
2	White roots and tubers	White potatoes, white yam, white cassava, or other foods made from roots	
3	Vitamin A rich vegetables and tubers	Pumpkin, carrot, squash, or sweet potato that are orange inside + other locally available vitamin A rich vegetables (e.g. Red sweet pepper)	
4	Dark green leafy vegetables	Dark green leafy vegetables, including wild forms + locally available vitamin A rich leaves such as amaranth, cassava leaves, kale, spinach	
5	Other vegetables	Other vegetables (e.g. Tomato, onion, eggplant) + other locally available vegetables	
6	Vitamin A rich fruits	Ripe mango, cantaloupe, apricot (fresh or dried), ripe papaya, dried peach, and 100% fruit juice made from these + other locally available vitamin A rich fruits	
7	Other fruits	Other fruits, including wild fruits and 100% fruit juice made from these	
8	Organ meat	Liver, kidney, heart or other organ meats or blood-based foods	
9	Flesh meats	Beef, pork, lamb, goat, rabbit, game, chicken, duck, other birds, insects	
10	Eggs	Eggs from chicken, duck, guinea fowl or any other egg	

11	Fish and seafood	Fresh or dried fish or shellfish	
12	Legumes, nuts and seeds	Dried beans, dried peas, lentils, nuts, seeds or foods made from these (eg. Hummus, peanut butter)	
13	Milk and milk products	Milk, cheese, yogurt or other milk products	
14	Oils and fats	Oil, fats or butter added to food or used for cooking	
15	Sweets	Sugar, honey, sweetened soda or sweetened juice drinks, sugary foods such as chocolates, candies, cookies and cakes	
16	Spices, condiments, beverages	Spices (black pepper, salt), condiments (soy sauce, hot sauce), coffee, tea, alcoholic beverages	

Part V: Household food insecurity access scale questions

1	In the past four weeks, did you worry that your household would not have enough food?	0= No……………………...<br>1=Yes	Q2
1.a.	How often did this happen?	1=Rarely (once or twice in the past four weeks)<br>2= Sometimes (three to ten times in the past four weeks)<br>3= Often (more than ten times in the past four weeks	
2	In past four weeks, were you or any HH member not able to eat the kinds of foods you preferred B/c of a lack of resources?	0=No…………………….<br>1=Yes	Q3

2.a.	How often did this happen?	1=Rarely<br>2= Sometimes<br>3= Often	
3	In past four weeks, did you or any HH member must eat a limited variety of foods you B/c of a lack of resources?	0=No………………………<br>1=Yes	Q4
3.a.	How often did this happen?	1=Rarely<br>2= Sometimes<br>3= Often	
4	In past four weeks, did you or any HH member must eat some food that really did not want to eat B/c of a lack of resources to obtain other types of food?	0= No……………………<br>1=Yes	Q5
4.a.	How often did this happen?	1=Rarely<br>2= Sometimes<br>3= Often	
5	In past four weeks, did you or any HH member must eat a smaller meal than you felt you needed B/c there was not enough food?	0=No………………………<br>1=Yes	Q6
5.a.	How often did this happen?	1=Rarely<br>2= Sometimes<br>3= Often	
6	In past four weeks, did you or any HH member must eat a fewer meals in a day's B/c there was not enough food?	0=No………………………<br>1=Yes	Q7

6.a.	How often did this happen?	1=Rarely<br>2= Sometimes<br>3= Often	
7	In past four weeks, was there ever no food to eat of any kind in your HH B/c of lack of resource to get food?	0=No………………………<br>1=Yes	Q8
7.a	How often did this happen?	1=Rarely<br>2= Sometimes<br>3= Often	
8	In past four weeks, did you or any HH member go to sleep at night hungry B/c there was not enough food?	0=No………………………<br>1=Yes	Q9
8.a.	How often did this happen?	1=Rarely<br>2= Sometimes<br>3= Often	
9	In past four weeks, did you or any HH member go to a whole day and night without eating anything B/c there was not enough food?	0 = No………………………<br>1=Yes	End
9.a.	How often did this happen?	1=Rarely<br>2= Sometimes<br>3= Often	

Part VI: Knowledge on nutrition

601	What is the quantity of food taken during pregnancy?	0. Less than or the same as before<br>1. More than before
602	How should a pregnant woman eat in comparison with a non-pregnant woman to provide good nutrition to her baby and help him grow?	0. Eat less food or energy<br>1. Eat more food (more energy)
603	Do you think that weight gain during pregnancy is normal process?	1. Yes<br>0. No
604	Do you think that under-nutrition during pregnancy is genetic origin?	1. Yes<br>0. No
605	Have you heard about under-nutrition during pregnancy?	1. Yes<br>0. No
606	Have you heard about iron-deficiency anaemia?	1. Yes<br>0. No
607	Have you heard about vitamin A deficiency during pregnancy?	1. Yes<br>0. No
608	Have you heard about vitamin B12 deficiency during pregnancy?	1. Yes<br>0. No
609	Do you think inadequate dietary intake and infection cause under-nutrition during pregnancy?	0. No<br>1. Yes
610	Do you think under-nutrition during pregnancy increase risk of complications?	0. No<br>1. Yes
611	Do you think under-nutrition during pregnancy increase risk of bad pregnancy outcomes?	0. No<br>1. Yes
612	Do you think under-nutrition during pregnancy increase risk of death of mother and new-born?	0. No<br>1. Yes
613	Do you think under-nutrition during pregnancy is communicable?	0. No<br>1. Yes
614	When a pregnant woman is undernourished, she is at risk of having a low-birth-weight baby, meaning that the baby is small or has a low birth weight.	0. No<br>1. Yes
615	Most women would benefit from major types of	0. No

	supplements, or tablets, during pregnancy.	1. Yes
616	Do you think under-nutrition during pregnancy can be prevented?	0. No<br>1. Yes
	**Part VII: Questions related to attitude on nutrition during pregnancy**	
701	How good do you believe it is to eat more food during pregnancy?	1. Not good at all<br>2. Not good<br>3. You're not sure<br>4. Good<br>5. Very good
702	How difficult do you feel about eating at least one additional meal during pregnancy?	1. Very difficult<br>2. Difficult<br>3. Neutral<br>4. Easy<br>5. Very easy
703	How likely do you feel you are to be iron-deficient/anaemic?	1.Not likely at all<br>2. Not likely<br>3. You're not sure<br>4. Likely<br>5. Very likely
704	How serious do you feel iron-deficiency/anaemia is?	2. Not likely at all<br>3. Not likely<br>3. You're not sure<br>4. Likely<br>5. Very likely
705	How good do you believe it is to prepare meals with iron-rich foods such as beef, chicken or liver?	1. Not good at all<br>2. Not good<br>3. You're not sure<br>4. Good<br>5. Very good
706	How difficult do you feel is it to prepare meals with iron-rich foods?	1. Very difficult<br>2. Difficult<br>3. Neutral

		4. Easy<br>5. Very easy
707	How confident do you feel in preparing meals with iron-rich foods?	1. Not confident at all<br>2. Not confident<br>3. Medium<br>4. Confident<br>5. Very confident
708	How likely do you feel to have lack of vitamin A in your body?	3. Not likely at all<br>4. Not likely<br>3. You're not sure<br>4. Likely<br>5. Very likely
709	How serious do you think a lack of vitamin A is?	1. Not very serious<br>2. Not serious<br>3. Neutral<br>4. Serious<br>5. Very serious
710	How much do you like the taste of the following foods?	**Liver** 1. Very dislike 2. Dislike 3. Neutral 4. Like 5. Very like<br>**Kidney** 1. Very dislike 2. Dislike 3. Neutral 4. Like 5. Very like<br>**Heart** 1. Very dislike 2. Dislike 3. Neutral 4. Like 5. Very like<br>**Egg** 1. Very dislike 2. Dislike 3. Neutral 4. Like 5. Very like<br>**Milk** 1. Very dislike 2. Dislike 3. Neutral 4. Like 5. Very like<br>**Cheese** 1. Very dislike 2. Dislike 3. Neutral 4. Like 5. Very like<br>**Yogurt** 1. Very dislike 2. Dislike 3. Neutral 4. Like 5. Very like<br>**Orange colored vegetables**<br>4. Very dislike 2. Dislike 3. Neutral

		4. Like 5. Very like<br>**Orange sweet potato**<br>5. Very dislike 2. Dislike 3. Neutral 4. Like 5. Very like<br>**Carrot** 1. Very dislike 2. Dislike 3. Neutral 4. Like 5. Very like<br>**Pumpkin** 1. Very dislike 2. Dislike 3. Neutral 4. Like 5. Very like<br>**Green vegetables**<br>1. Very dislike 2. Dislike 3. Neutral 4. Like 5. Very like
711	How serious do you feel a lack of iodine in the body is?	1. Not very serious<br>2. Not serious<br>3. Neutral<br>4. Serious<br>5. Very serious
712	How good do you feel it is to prepare meals with iodized salt?	1. Not good at all<br>2. Not good<br>3. You’re not sure<br>4. Good<br>5. Very good
713	How difficult is it for you to buy and use iodized salt?	6. Very difficult<br>7. Difficult<br>3. Neutral<br>4. Easy<br>5. Very easy
714	How likely do you feel you are to be under-nutrition during pregnancy?	1. Not likely at all<br>2. Not likely<br>3. You’re not sure<br>4. Likely<br>5. Very likely
715	How serious do you feel under-nutrition during pregnancy is?	1. Not likely at all<br>2. Not likely

		3. You’re not sure<br>4. Likely<br>5. Very likely
